# Unveiling Texture and Topography of Fatty Acid Langmuir Films: Domain Stability and Isotherm Analysis

**DOI:** 10.1021/acs.langmuir.3c03501

**Published:** 2024-05-07

**Authors:** Erik Bergendal, Mark W. Rutland

**Affiliations:** † Department of Chemistry, School of Engineering Sciences in Chemistry, Biotechnology and Health, KTH Royal Institute of Technology, Teknikringen 30, Stockholm SE-100 44, Sweden; ‡ RISE Research Institutes of Sweden, Chemistry, Materials and Surfaces, Box 5607, Stockholm SE-114 86, Sweden; § School of Chemistry, University of New South Wales, Sydney, New South Wales 2052, Australia; ∥ Laboratoire de Tribologie et Dynamique des Systèmes, École Centrale de Lyon, Ecully Cedex 69134, France

## Abstract

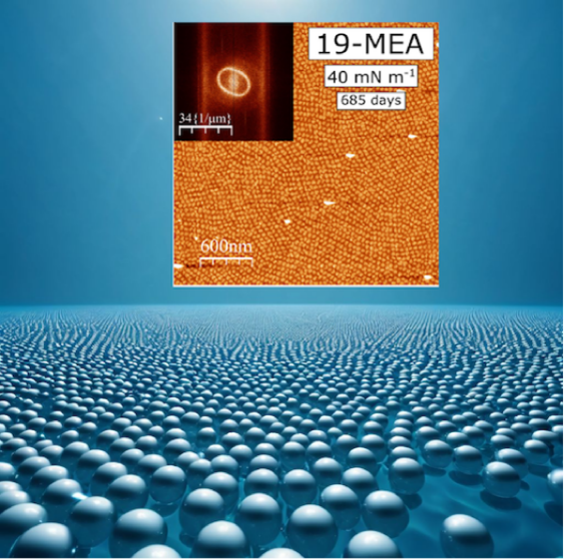

3D texturing by self-assembly at the air–water interface has recently been proposed. The hypothesis of this work is that, if this is true, such domain formation should be inferable directly from pressure–area isotherms and be thermodynamically stable. Monolayers of branched fatty acid mixtures with straight chain analogues and their stability are thus studied using a combination of pressure–area isotherms, thermodynamic analysis, *in situ* Brewster angle microscopy, and atomic force microscopy of both LB-deposited and drop-cast films on silicon wafers. Isotherms reflecting the behavior of monodisperse 3D domains are shown to be independent of compression rate and display long-term stability. Gibbs analysis further confirms the thermodynamic rather than kinetic origin of such novel species by revealing that deviations from ideal mixing can be explained only *a priori* by differences in the topography of the water surface, thus also indirectly confirming the self-assembly deformation of the water interface. The intrinsic self-assembly curvature and miscibility of the two fatty acids is confirmed by drop-casting, which also provides a rapid, tunable thin-film preparation approach. Finally, the longevity of the nanostructured films is extraordinary, the long-range order of the deposited films increases with equilibration time at the water interface, and the integrity of the nanopatterns remains intact on the scale of years.

## Introduction

Floating monolayers of long chain fatty acids have been studied at the air–water interface for more than a century,^1,2^ and have been thoroughly investigated in terms of phase behavior,^3−7^ molecular packing,^8−10^ monolayer stability,^11−14^ and ion-specific interactions with subphase electrolytes.^15,16^ Confined by insolubility to the air–water interface, surfactant monolayers are thermodynamically treated as two-dimensional systems (disregarding the molecular length of the surfactant) and commonly characterized by pressure–area isotherms. While this is very much a macroscopic measurement, such isotherms lend themselves to detailed interpretation of molecular interactions. On compression, changes in the isotherm shape are attributed to the monolayer undergoing one or more phase transitions, depending on the surfactant,^17^ subphase pH,^7,18^ temperature,^15^ and nature of solvated salts.^5,19^ Analogous to phase transitions in three dimensions, a first-order phase transition observed in a pressure–area isotherm of floating monolayers would be expected to occur at a constant pressure. However, this was early observed not to be the case, which was suggested to be an indication of the coexistence of surface monolayer regions (patches) in the phase transition region.^20,21^ A large body of work was concerned with predicting equations of state for phase transitions in an array of floating monolayers while taking the presence of ordered structures of surfactants into account, where two-dimensional, “cartwheel” like micelles were first touted.^22−24^ The presence of floating patches on the micrometer scale was observed *in situ* with fluorescence microscopy^25−27^ and Brewster angle microscopy (BAM).^15,28−31^ These are thus much larger than the predicted surface micelles, which would have a diameter of roughly two molecular lengths.^22^

By using the Langmuir–Blodgett (LB) technique, floating monolayers can be transferred from the air–water interface to a solid support.^32,33^ The transferred film can then be characterized by atomic force microscopy (AFM) for high resolution imaging, which is unfeasible at the air–water interface.^34,35^ In this manner, structures intermediate between the microscopic patches and micellar cartwheels have been observed in systems consisting of low molecular weight amphiphiles, in particular semifluorinated alkanes. These domains at the air–water interface, are described as monodisperse circular structures and elongated worm-like structures, depending on the amphiphile molecular structure.^36−40^

Singly methyl-branched, long chain fatty acids also display domain formation at the air–water interface, which systematically vary in size and shape with both methyl branch position,^41,42^ and the ratio between branched and analogous unbranched fatty acids in mixtures.^43^ Such branched chain fatty acid domains have furthermore, uniquely, been observed to texture the underlying water surface three-dimensionally, in order to accommodate the packing constraints of the hydrocarbon chain, induced by the methyl branch.^42,43^ That is, the water interface itself bends locally to accommodate the self-assembly curvature.

Although such domain-forming films have so far been well characterized at the air–water interface and as deposited films, thorough analysis and interpretation of pressure–area isotherms of domain-forming films has been lacking. So far, such analysis has been carried out in classical terms of “liquid” and “solid condensed” monolayer phases, terminology used to classify monolayer tilt and disorder in 2D films.^6,36,44^ However, in a floating film consisting of well-ordered domains, such classification loses its meaning. Here, an effort has been made to interpret pressure–isotherm data, in terms of Gibbs excess free energy of mixed monolayers^45,46^ for the branched fatty acid 18-methyleicosanoic acid (18-MEA) and its straight chain analogue eicosanoic acid (EA), while taking the 3D texturing properties of domain-forming films into account.^47−52^ Furthermore, the stability of these domain-forming films has been investigated at the air–water interface, as well as on solid supports as deposited monolayers.

## Experimental Section

All isotherms were collected using a KSV Nima 5000 Langmuir trough from Biolin Scientific. The trough was made of polytetrafluoroethylene (PTFE) with an effective water area of 755.25 cm^2^. Symmetric compression of the polyoxymethylene (POM) barriers was performed at 9 cm^2^ min^–1^, unless stated otherwise. The trough and barriers were cleaned by rinsing with absolute ethanol, followed by thorough rinsing with Milli-Q water (>18.2 MΩ cm and <2 ppm total organic compounds). Prior to rinsing, the trough was wiped with lint-free paper soaked in chloroform. Paper Wilhelmy plates were used to monitor surface pressure to an accuracy of 0.01 mN m^–1^, and a circulation bath was used to control the water temperature to 22.0 ± 0.1 °C. Monolayers were spread from a 1 mg mL^–1^ fatty acid solution in chloroform. After spreading, the chloroform was allowed to evaporate for 15 min before barrier compression. LB depositions were performed at 1 mm min^–1^. A 10 min waiting time was allowed before depositions upon reaching the target pressure. All depositions were monolayer depositions performed on the upstroke at 20 mN m^–1^ unless stated otherwise. The selected pressure of deposition was chosen for comparison between systems at a pressure far from the system collapse pressure. The constant surface pressure was maintained by continuous barrier compression.

Eicosanoic acid (Sigma-Aldrich, ≥ 99%), 18-methyleicosanoic acid (Larodan, > 99%), cadmium chloride (Fluka, > 99%), sodium hydrogen carbonate (Merck, 99.7%), and hydrochloric acid (Fluka, 4 M in H_2_O), were used as received. All fatty acid solutions were prepared to 1 mg mL^–1^ in chloroform (Merck, 99.0–99.4% GC, approximately 1% ethanol as stabilizer).

Drop casts and LB depositions were made on low roughness (<2 Å) silicon wafers. Prior to sample preparation, the wafers were sonicated in absolute ethanol for 15 min, followed by rinsing with Milli-Q water. Subsequently, the wafers were immersed in chromosulfuric acid (Merck, ≥ 92% H_2_SO_4_, ≥ 1.3% CrO_3_) for 5 min, followed by thorough rinsing with Milli-Q water.

AFM measurements were carried out on a Multimode Microscope 8 (Bruker), using silicon cantilevers (MikroMasch, HQ:NSC35/AL BS) with nominal stiffness and resonant frequency of 5.4 N m^–1^ and 150 kHz, respectively. Prior to use, cantilevers were subjected to UV-radiation for 10 min. AFM images were analyzed using the WSXM software.^75^

BAM images of fatty acid monolayers at the air–water interface were collected at 53.1° to the surface normal using a Nanofilm EP3 (Accurion) equipped with a Nd:YAG (532 nm) laser, and a 10× objective. Fast-focusing capture was used to stick together several images in focus of the mobile monolayers captured at low surface pressure (<0.5 mN m^–1^). No background subtraction was performed for any of the presented images.

## Results and Discussion

**Figure 1:**
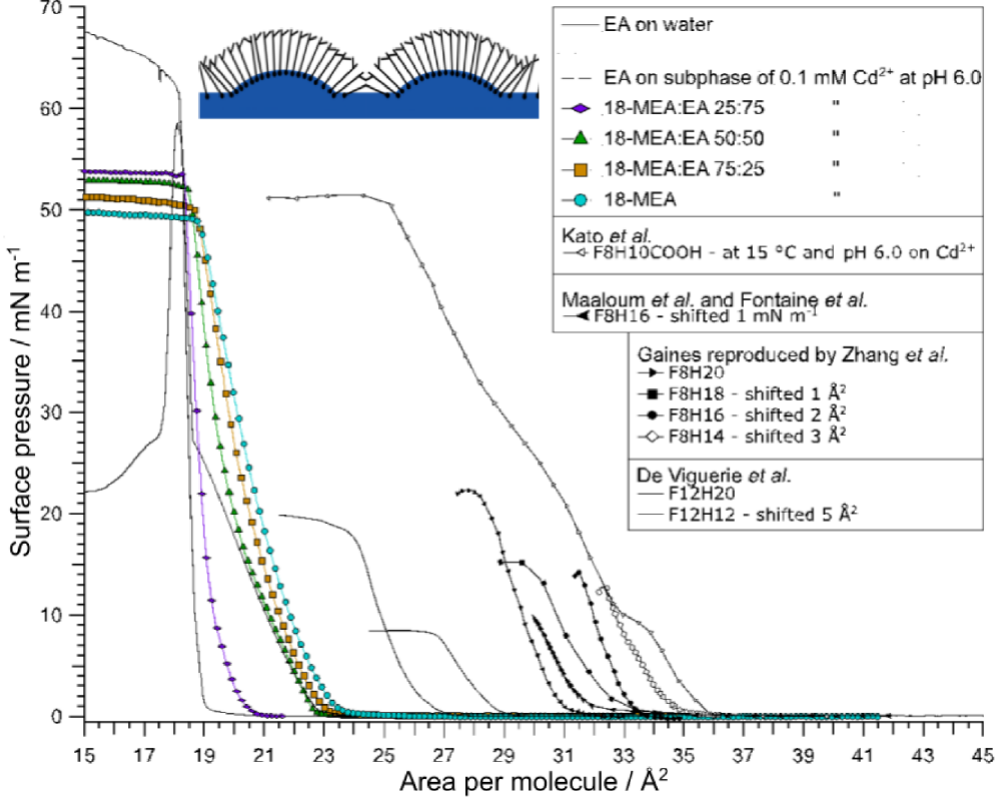
Colored symbols: Langmuir isotherms were performed on a Cd^2+^ subphase at pH 6.0 on films of the saturated long chain fatty acids EA (dashed line) and 18-MEA (circles) and mixtures of the two in the ratios 18-MEA:EA 75:25 (squares), 50:50 (triangles), and 25:75 (diamonds). An isotherm of EA (solid line) on a neat water subphase is added for reference. Monolayers were compressed at 9.0 cm^2^ and min^–1^. In addition, isotherms for a range of domain-forming substances have been adapted from the references shown in the respective figure legend, with rights and permissions detailed in the ESI. Isotherms have been shifted to allow comparison of both slope and collapse pressure.

The isotherm behavior of novel self-assembly structures recently reported as deforming the water interface out of plane into a “cobbled” structure,^42,43^ is first examined. Figure 1 shows isotherms of mixed monolayers of EA and 18-MEA, as well as the two pure cases. The self-assembly curvature that induces the water bending arises from the fact that the area of the terminal region of the branched alkyl chain is larger than the area occupied by the headgroup. This is due to the presence of a divalent cation (in this case, Cd^2+^, buffered to pH 6) in the subphase, which reduces the electrostatic interactions. The behavior of Langmuir monolayers and films deposited from 18-MEA and EA, on a *pure water* subphase behave classically; they do not show monodisperse domains but rather solid- and liquid-like phase behavior, since the headgroup area is larger, and the inherent curvature imposed by the area mismatch between head and tail disappears. The isotherms, AFM images, and BAM images of these films are presented and discussed in the ESI (Figures S1–S3).

All the isotherms containing 50% 18-MEA or more lack the well-defined features corresponding to the untilted condensed 2D phase displayed by the straight EA chain. Furthermore, the collapse behavior is characteristic of constant pressure, unlike the unbranched analogue’s collapse at constant area. The rather different behavior, with the larger change in apparent area for the 18-MEA, is thus held to be due to the presence of nanodomains where the water surface is curved to accommodate the self-assembly packing. (Note that both the pressure at which the films collapse, and the area at which liftoff occurs, vary systematically with the proportion of branched fatty acid. The branched fatty acid has a larger molecular cross-section, and therefore the liftoff occurs at larger apparent area. Due to the different geometries of the domains at the different compositions, however, the systematic liftoff variation is nonlinear with the composition.)

Previous accounts of low molecular weight surfactants forming monodisperse domains in the 50 nanometer-range at the air–water interface, have in common that little to no headgroup interaction is present;^36−40,42,53^ This has either been due to the lack of a hydrophilic headgroup, such as the case for partially fluorinated alkanes, or due to headgroup charge screening via controlled subphase salt and pH conditions,^36,41,42^ While the monodisperse domains have been documented, explanations for the existence of the domains, which have uniformly invoked a 2D arrangement on a flat water interface, have remained elusive. As a qualitative comparison, pressure–area isotherms of several domain-forming surfactants from the literature have been recreated in Figure 1, together with isotherms of 18-MEA, EA, and mixtures of the two fatty acids, collected on a 0.1 mM Cd^2+^ subphase at pH 6.0.

The addition of salt to the subphase of fatty acid monolayers screens the charges between dissociated carboxyl headgroups, leading to a different carboxylate–counterion interaction depending on pH and the nature of the counterions. Cadmium(II) is known to not only screen electrostatic charges, but also bind covalently to the carboxylate moiety, effectively condensing fatty acid monolayers.^54−56^ This is clearly exemplified by the isotherm of EA (dashed black line without markers in Figure 1), which directly enters an untilted condensed phase upon isotherm liftoff.^6,57^ Deposited monolayers of EA under these subphase conditions show featureless, homogeneous monolayers with orthorhombic chain packing.^34,41^ The isotherms of 18-MEA:EA monolayers on the cadmium subphase show what would classically be interpreted as a “tilted condensed” phase upon liftoff, with an increasing fracture collapse pressure with an increasing EA fraction in the monolayer. Deposited films of 18-MEA and its mixtures with EA have been observed to generate a series of surface morphologies—analogous to bulk lyotropic liquid crystals—consistent with a self-assembly induced curvature provided by the branched methyl chain.^42,43,58^ It is noticeable from Figure 1, that the isotherms of the domain-forming 18-MEA:EA mixtures show similar liftoff behavior and slopes as the adapted isotherms of surfactants forming analogous domains.^36−40,53^ While the maximum value of the pressure–area isotherms varies in terms of surface pressure, due to the presence or lack of a hydrophilic headgroup, the overall behavior is highly reminiscent. Recent neutron reflectometry (NR) measurements in the case of branched fatty acids^42,43^ indicate that the water interface increases in topography as the compression increases in this region, and thus that conventional, 2D phase descriptions of Langmuir films are inappropriate to describe the systems. The literature isotherms also lack the features that correspond to condensation of 2D phases, also collapse at constant pressure, and are highly similar in their characteristics to those of the fatty acids, where a topography of the water interface is now established. (The isotherms tend to increase in gradient gradually, rather than displaying the abrupt gradient changes associated with phase transitions, and the linear evolution of the solid condensed phase.) It would thus seem likely that, when the headgroup repulsion constraint is relaxed, such an isotherm shape could be used as an indicator of domain-formation at the air–water interface and, as a corollary, that a self-assembly curvature phenomenon is in fact responsible for the domain texture in all the previous cases of such ordered domains.

## Domain Stability and the Stability of Domain-Forming Films

Such water structuring, domain-forming monolayers are still rare and require more careful exploration from a pressure–area isotherm perspective. While the monodispersity and size both suggest a thermodynamic (self-assembly) rather than a kinetic mechanism,^41−43^ it remains to be seen how stable such domains are, whether there are dynamic responses to consider, or indeed different collapse mechanisms. The stability at the air–water and air–solid interfaces is thus now addressed.

**Figure 2:**
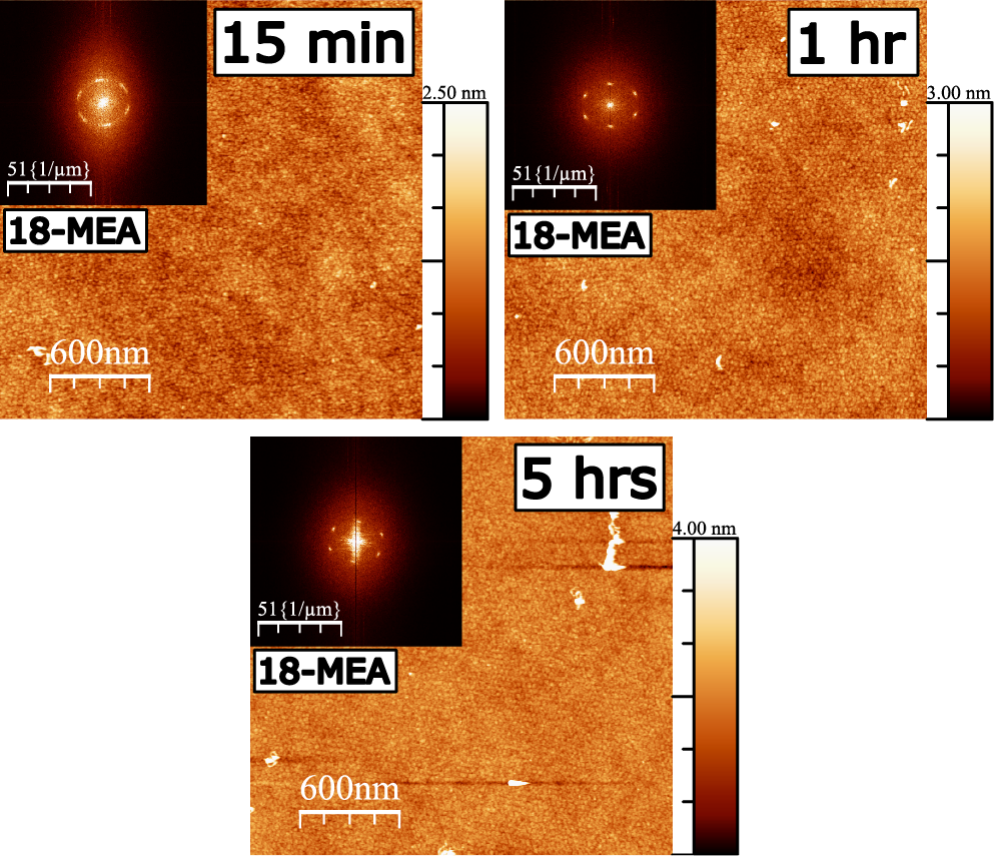
AFM height images of deposited monolayers of 18-MEA on a silicon wafer, deposited at 20 mN m^–1^ from a 0.1 mM Cd^2+^ subphase at pH 6.0. The monolayer stabilization time before deposition is indicated in the top right corner. FT insets show an increased ordering with stabilization time, which is analyzed with azimuthal integration and shown in detail in Figure S4.

First, to address the longevity of the domains and their ordering at the air–water interface at a given pressure, a series of depositions were performed from the same monolayer, under the same conditions, but after different waiting times. AFM height images of these depositions are presented in Figure 2, where the time of deposition is indicated in the top right of each image as the time after the target deposition pressure of 20 mN m^–1^ was reached for the monolayer. In every case, domains of roughly 32 nm are observed, consistent with earlier studies using these molecules.^41,43^ Larger images are shown to provide statistics for the Fourier transform (FT) analysis, as an inset for each image. There, the distance from the center indirectly provides the domain size, and the hexagonal pattern indicates domain ordering. The intensity observed in the middle of the FT of the AFM of the monolayer deposited after 5 h is due to the appearance of regions of multilayers (nucleation and growth, see later) at the air–water interface producing comparably large (observed close to FT center) and high (bright on AFM image) domains. With increasing stabilization time before deposition, the hexagonal ordering of the surface domains is further refined, as seen by the narrowing of the intensity signal. This increased hexagonal ordering of the domains is analyzed by azimuthal integration of the FT intensity, where a narrowing of the fwhm is observed with increasing stabilization time, as depicted in Figure S4. This consolidation of the ordering combined with a slow multilayer formation would be expected to impact the isotherm stability, and is addressed in an ensuing section. Finally, as domain ordering and rearrangement occur without coalescence over time, the hypothesis of a thermodynamically predetermined 3D domain size is strongly supported.^43^

**Figure 3:**
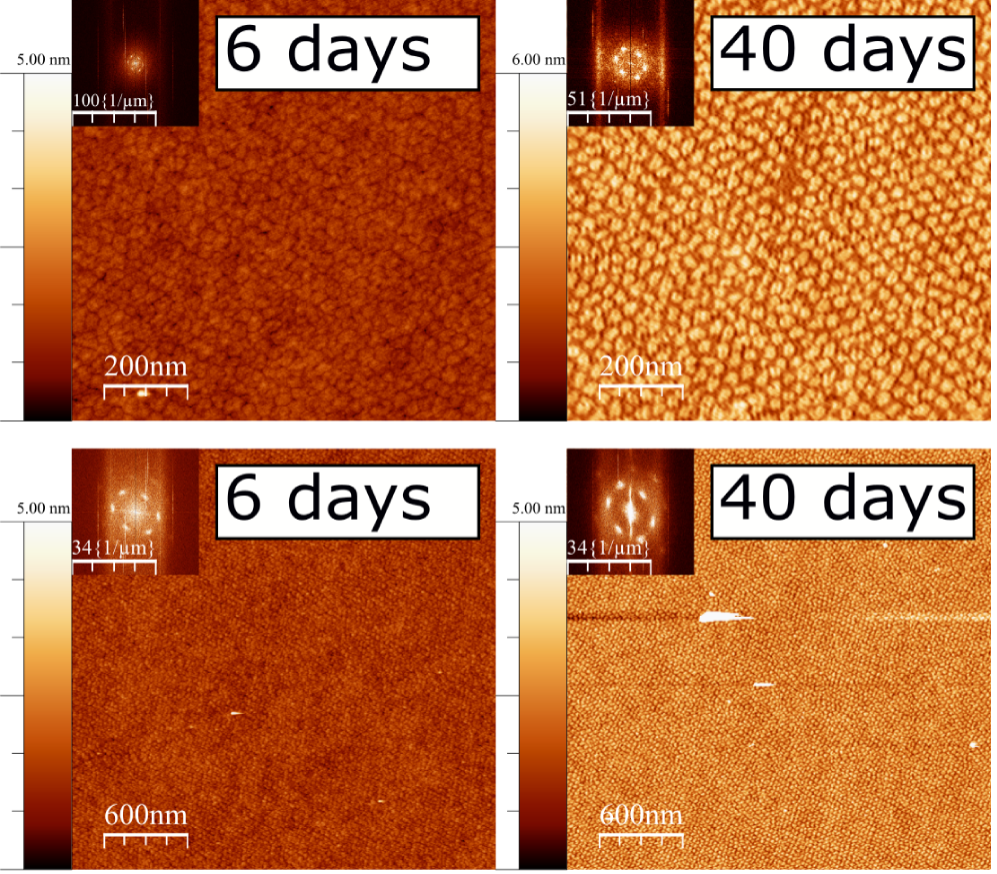
AFM height images of 18-MEA monolayers deposited on silicon wafers at 20 mN m^–1^, at magnifications of 1 × 1 μm^2^ (top, to allow the domain morphology to be observed) and 3 × 3 μm^2^ (bottom, to demonstrate long-range nature of the patterns). Images were taken 6 (left) and 40 days (right) after monolayer deposition. Samples were stored in airtight containers in ambient conditions. FT inserts show that the hexagonal ordering of the monodisperse domains is retained after long-term storage. Note that the images after 6 and 40 days are not of the same area as the monolayer.

As an extension of this study and as an attempt to investigate the potential of these monolayers for nanopatterning of surfaces, the stability of the deposited layers on the solid surface was also studied as a function of storage time after deposition. A monolayer of 18-MEA, deposited on a silicon wafer from a 0.1 mM Cd^2+^ subphase at pH 6.0, was stored in an airtight container at ambient conditions prior to AFM measurements. The same monolayer was imaged 6 days after deposition and then again 40 days after deposition, as presented in Figure 3 to the left and right, respectively. A well-packed monolayer of monodisperse domains of hexagonal ordering was observed 6 days after deposition. 40 days after deposition, similar hexagonal ordering was observed; however, the close-packing of the domains had relaxed, indicating that the thermodynamically stable domain-size at the air–water interface is not maintained indefinitely at the air–solid interface. The domain sizes along the short and long axes are determined from FT insets to 32 and 42 nm, respectively, for AFM images 6 days after deposition and 26 and 36 nm, after 40 days. This is reasonable if one considers that a 3D textured film of spherical caps on a deformable air–water interface is transferred to a rigid 2D silicon wafer. Importantly, the integrity of the domains was maintained; no domain coalescence is observed over time, indicating that molecular rearrangements are confined to separate domains, which suggests a preferred domain size of 18-MEA at the air–solid interface. The restructuring may be affected by the evaporation of water, remaining either as a thin film during deposition or as the hydration of the headgroup ions. As an additional test, a deposited film from an earlier study^42^ was examined 2 years after deposition, and the pattern was found to be intact, as shown in Figure S5.

**Figure 4:**
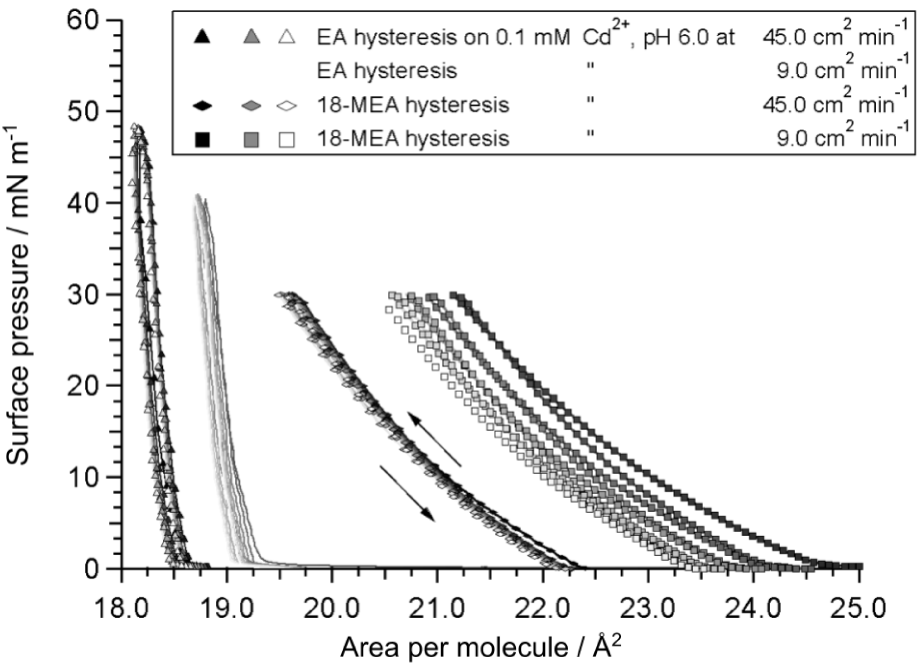
Hysteresis isotherms for monolayers of EA and 18-MEA at two different compression rates. (i) 9.0 cm^2^ min^–1^ (EA: circles, 18-MEA: squares) and (ii) 45 cm^2^ min^–1^ (EA: triangles, 18-MEA: diamonds). The isotherms were reversed below the respective collapse pressures of the systems. Five and seven compression–expansion cycles were performed for the 18-MEA and EA isotherms, respectively. To facilitate comparison, the isotherms have been arbitrarily shifted along the *x*-axis.

To address the elasticity of the films at the air–water interface and obtain additional information on their properties and stability, hysteresis isotherms were performed and are presented in Figure 4. Compression–expansion cycles of monolayers of EA (no domains) and 18-MEA (clear domains) were performed at two different compression rates up to a pressure below their respective fracture collapse pressure. Little to no hysteresis was observed in the monolayers, and no difference in hysteresis was observed for the different compression rates. The shift toward lower areas per molecule for the slower compression speeds is due to continuous monolayer collapse, rather than rate dependent effects. The lack of hysteresis indicates that compression of the domain film is reversible and that any viscoelastic domain or molecular rearrangements upon compression are on a time scale not observable in such experiments. It also dispels the remote possibility that the domains might in some way have been an artifact of compression rate.

**Figure 5:**
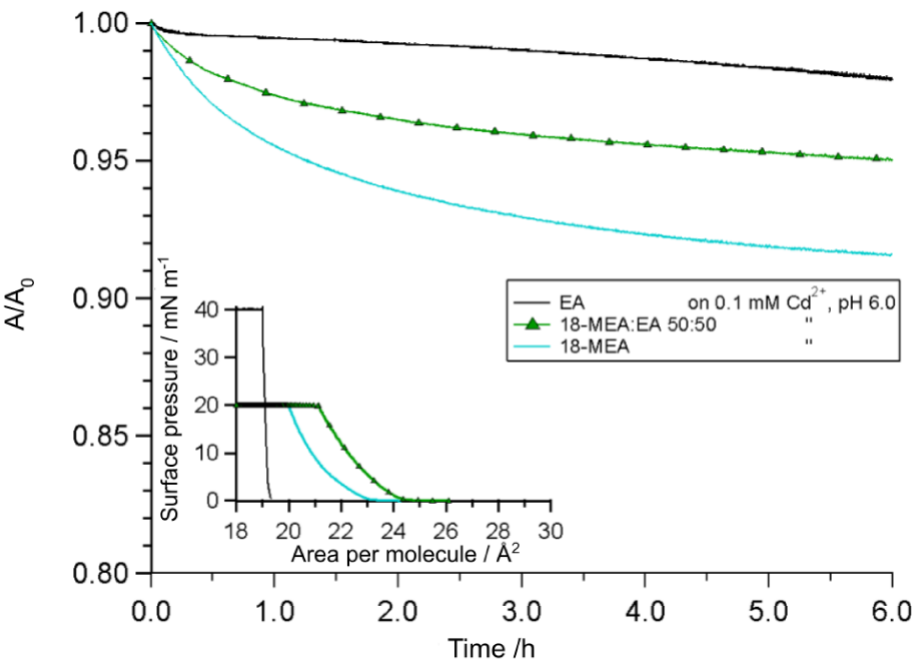
Isotherm stability as a function of time for EA (black line), 18-MEA (blue line, lowest), and 18-MEA:EA 50:50 (triangles). The fractional reduction in area was measured while maintaining a constant surface pressure of 20 mN m^–1^ for 18-MEA and 18-MEA:EA 50:50, and 40 mN m^–1^ for EA. The isotherms with their target pressures are shown in the inset to the bottom left.

At any surface pressure above the equilibrium spreading pressure,^59−61^ a monolayer is known to undergo continuous collapse following nucleation and growth theory.^13,62^ This phenomenon can be observed as monolayer instability, manifested as a reduction in area for a monolayer kept at constant pressure or a reduction in pressure for an experiment performed at constant area. Figure 5 shows the stability in area (normalized by initial area) versus time for monolayers of EA at 40 mN m^–1^ and 18-MEA and 18-MEA:EA 50:50 at 20 mN m^–1^, on a subphase of 0.1 mM Cd^2+^ at pH 6.0. Over the course of 6 h, around 2% area decrease was observed for the monolayer of EA, where the rapid initial decay is attributed to molecular rearrangements in the flat monolayer.^11,60,63^ The area decay observed for 18-MEA (10%) and the mixed system (5%) displays a more rapid initial area loss, which leveled out after several hours. The larger, though still relatively small^11^ area reduction in the case of the two monolayers containing 18-MEA, is explained by the consolidation of domain order (in addition to any molecular rearrangements analogously to EA). Such a consolidation of domain ordering is clearly observed in the FT inset of Figure 2. This implies that the area loss corresponds to gaps between imperfectly ordered domains, which achieve an improved hexagonal close packing when compressed over a period of hours.

**Figure 6:**
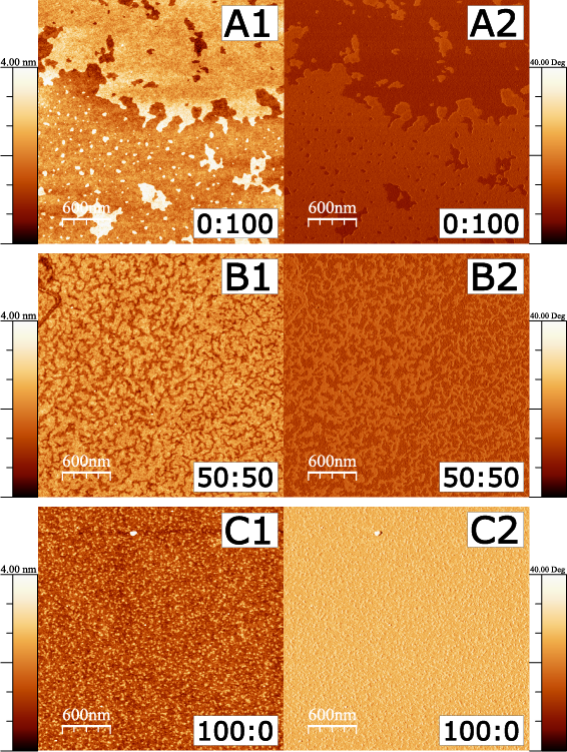
AFM height (left) and phase (right) images of monolayers formed by drop-casting EA, 18-MEA:EA 50:50, and 18-MEA on dry silicon wafers. Drop casts of EA show a patchy albeit homogeneous monolayer. A dendritic structure is observed for the mixed monolayer, and 18-MEA shows domain formation with a characteristic size and domain distance.

Drop casting on dry silicon wafers was performed to investigate the self-assembly behavior of 18-MEA at the air–solid interface and was subsequently investigated with AFM. There is a 3-fold point to this exercise. First, we need to see whether there is any evidence for phase separation in mixed systems. Second, we investigated whether there is an intrinsic difference in the behavior of the branched and straight chain molecules, even in the absence of cadmium ions and a deformable interface. And third, to discount the remote possibility that the domains formed in the AFM images are a result of self-assembly induced by a solid substrate and not the deformable air–water interface. Resulting images are presented for EA, 18-MEA:EA 50:50, and 18-MEA from A–C in Figure 6, with height and phase modulation images denoted 1 and 2, and shown to the left and right, respectively. Drop casts of EA display a homogeneous albeit patchy monolayer with a height of 2.4 nm (as determined from line profiles presented for all drop casts in Figure S6) with sharp edges toward the underlying substrate. The monolayer height corresponds well to the extended chain length of a C_20_ hydrocarbon chain.^58,64^ The mixed system shows an unordered network with dendritic shapes and a height modulation of roughly 1.5 nm. Drop casts of 18-MEA show sparse coverage of domains roughly 30–50 nm in size and with a domain height around 2 nm. All drop casts thus show distinctly different monolayer patterns, and the observation that the mixed monolayer does not reflect the patterns of either EA or 18-MEA further indicates a complete mixing of the two fatty acids. The domain formation by 18-MEA additionally confirms that an intrinsic property of 18-MEA is to not form monolayers in the classical sense of homogeneous flat films, which is a result of the chain packing constraints by the methyl branch. As a result of the high concentration of the chloroform solution, large micrometer-sized crystallites are formed in ordered arrays on the silicon wafer. These are shown in Figure S7 by optical microscopy and in Figures S8 and S9 as images by AFM. The high resolution AFM images in Figure 6 show representative images of the monolayer-like regions between the larger crystallites.

## Isotherm Analysis

There are various thermodynamic tools that can be employed to address pressure–area isotherms, particularly for mixed monolayer systems. These approaches assume, however, that the film at the air–water interface consists of a 2D monolayer, unlike the domain-forming films of 18-MEA. In order to examine the hypothesis made above (*i.e.*, that the isotherm shape is an indicative characteristic of domain formation, with its associated topographic deformation), a thermodynamic investigation of the 18-MEA:EA system follows.

The integrated area under a pressure–area isotherm provides an estimation of the change in Gibbs free energy of the system by
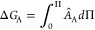
1where *Â* is the area per molecule in Å^2^ and Π is the surface pressure. The expected change in excess free energy for a mixed system can thus be estimated by a linear combination of that of the pure compounds^45,46,65,66^
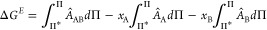
2where *x*_A_ and *x*_B_ are fractions of components A and B, respectively. The surface pressure Π* denotes a surface pressure at which components A and B are assumed to mix ideally in the monolayer, and has been chosen as 0.3 mN m^–1^ for monolayers of 18-MEA:EA, based on the absence of a liquid expanded phase at large areas per molecule. From eq 2, ideal mixing in a two-component system should result in a zero excess free energy, which would signify either mixing without an area change or complete phase separation. Neither the BAM images, nor the images of the deposited films^43^ suggest that there is phase separation. BAM images for EA, 18-MEA, and the mixed monolayers on 0.1 mM Cd^2+^ subphase at pH 6.0 are presented in Figures S10 and S11, together with AFM images from ref (43) of deposited monolayers of the same fatty acid compositions in Figure S12. Negative deviation from ideal behavior in the excess free energy signifies a reduced energy of the system due to attractive mixing, and the opposite implies repulsive mixing.

**Figure 7:**
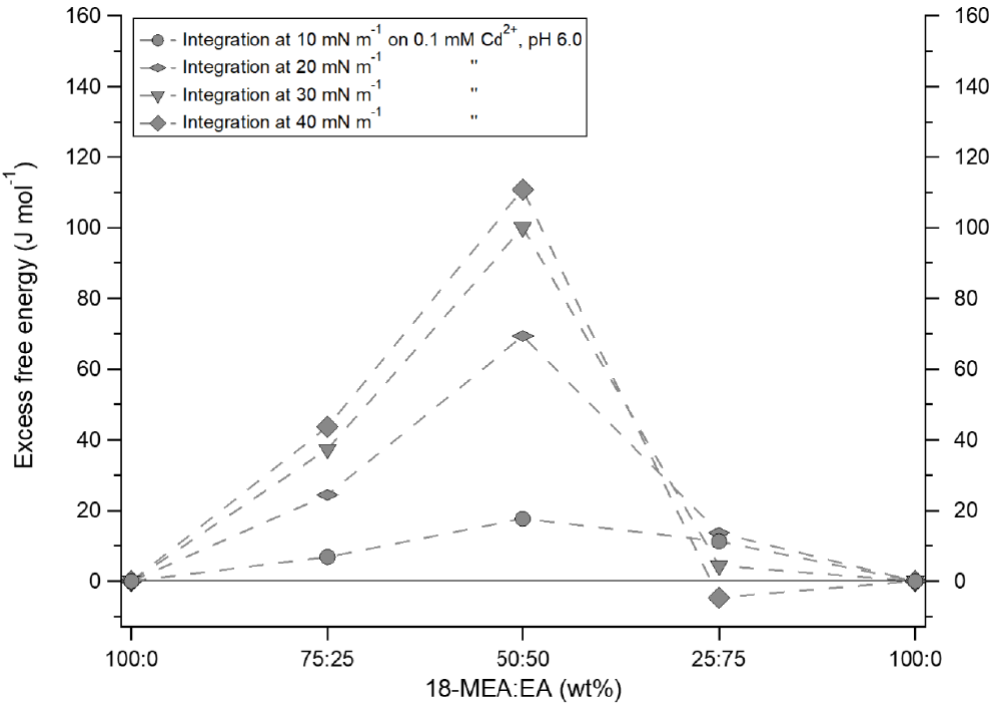
Gibbs excess free energy for mixed monolayers of EA and 18-MEA on a 0.1 mM Cd^2+^ subphase at pH 6.0. Energies have been calculated at distinct surface pressures based on the isotherms shown in Figure 1. The dashed lines are guides for the eye.

Calculated excess free energy for mixtures of 18-MEA:EA, using the isotherms from Figure 1 are summarized in Figure 7, for four different surface pressures. A positive excess free energy is observed for 75:25 and 50:50, which increases with surface pressure, whereas the opposite is observed for 27:75, indicative of repulsive mixing for the former ratios, from a classical analysis. This would seem unlikely considering the previously reported systematic domain formation for these fractions, and the distinct pattern observed by drop casting, which would rather indicate an ideal or attractive mixing. This result is thus difficult to interpret from a classical thermodynamic analysis in terms of the 2D model (surface pressure versus area per molecule) for which such an analysis is designed. However, if one accepts that the isotherms are in fact the reflection of an increased 3D texturing of the interface with increasing pressure, then the difference in “excess free energy” can only be interpreted in terms of the additional surface area and gravitational work associated with the different geometries of the domains under different curvature conditions (EA fraction). Based on the previously reported domain sizes for the 18-MEA:EA 75:25 and 50:50 mixtures, and their inferred domain heights from neutron reflectometry at the air–water interface, an increased surface area due to domain formation can be estimated. This area increase corresponds to a change in total surface energy, which for the 75:25 and 50:50 mixtures relative to 18-MEA, results in an increased excess free energy of 25 and 81 J mol ^–1^, respectively, with calculations further detailed in the ESI (the higher EA-fraction was not included as it does not form domains at the air–water interface, see Figure S12).^43^ For such a simple calculation, the correlation between a geometrical estimation and experimental data is striking, and strengthens greatly the thesis that the pressure–area isotherm can indeed be used as a tool to reveal surface domain formation.

Finally, monolayer isotherms can be characterized by the compressional modulus
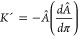
3where *Â* and π are the area per molecule and surface pressure from the isotherm, respectively. The compressional moduli have been calculated from isotherms of EA, 18-MEA, and mixed systems collected at a 0.1 mM Cd^2+^ subphase at pH 6.0, and are presented in Figure S13, where systematic variation with the fraction of straight chain is once again observed. A large difference between films with “monolayer” characteristics as opposed to “self-assembly nanodomain structure” is observed. The compressibility of monolayers of 18-MEA, 75:25, and 50:50 show a compressional modulus between 100 and 500 mN m^–1^, which according to classical monolayer analysis of straight chain saturated fatty acids would be considered indicative of a “liquid condensed” phase.^67^ By the same analysis, monolayers of EA and 25:75 would be considered to yield a high compressional modulus even when compared to values reported to correspond to a “solid” phase (which is likely due to the condensation of the monolayer by the Cd-ions, compared to reported accounts measured on water).^67^ This analysis, however, again assumes a flat monolayer, with an in-plane homogeneous molecular tilt of the hydrocarbon chain, decreasing with increasing surface pressure. As this is not the case for the domain-forming 18-MEA (and its high 18-MEA fraction mixtures with EA) on a Cd^2+^ subphase, the observed compressional modulus is rather an indication of compressibility of an assembly of surface domains. This is in agreement with the high compressional modulus of the high EA-fraction monolayer 25:75, where only large grains have been observed, rather than monodisperse surface domains, and the monolayer indeed does show distinct untilted condensed (or “solid”) behavior.^43^

## Conclusions

Packing constraints force the branched monolayer films into three-dimensional aggregates, imposing a topography on the water surface. This occurs when the headgroup area is constrained and is sufficiently small that the methyl branch hinders a close packing of the alkyl chains. Such a close packing would be experienced by the unbranched eicosanoic acid when the headgroup charge repulsion is screened by electrolyte or when metal ion–headgroup covalent character is present.

On the other hand, when headgroup interactions dominate the self-assembly at a pure water surface, branched fatty acids do not experience such a curvature imperative. Under these conditions, the behavior and the isotherms can be described by the classical 2D approach, which has previously characterized all studies of Langmuir films. While the isotherms do not display obvious features that can be directly attributed to topography, it is nonetheless shown that it is possible to distinguish between the 2D and 3D cases directly from the isotherm behavior. Rather it is the absence of features such as phase transitions that reveal the self-assembly water surface structuring. Comparison with earlier published studies of “circular” aggregates—in partially fluorinated alkane systems where there is also a mismatch in cross section between different regions of the alkane chains—shows that this isotherm character appears to be a general feature.^36−40,53^ The hypothesis is thus advanced that the monodisperse, 2D domains in those systems are also the result of self-assembly-induced curvature and that rather than being circular, they are likely spherical caps.

Thermodynamic analysis in terms of the excess Gibbs free energy for the mixed systems reveals behavior that cannot be explained in terms of mixing in a 2D monolayer. The approach has previously been successfully used to characterize non domain-forming monolayers of straight chain fatty acids, fatty alcohols, and phospholipid monolayers.^46,68,69^ The analysis can only be reconciled if the different surface areas associated with the different aggregate geometries are considered. Then the magnitudes of the apparent free energy differences can be explained simply in terms of the difference in the total surface energy. As a corollary, it would appear that pressure–area isotherms are also a useful means of extracting the work required to form the additional surface area associated with the 3D texturing, thus extending the usefulness of such analysis.

Finally, the stability of the aggregates at both the air–water and air–solid surfaces is remarkable. Such stability is unusual for adsorbed monolayers, which generally collapse to multilayers upon prolonged storage,^70−72^ or require postdeposition polymerization to increase stability.^73,74^ Consolidation of the domains at the air–water interface is, unsurprisingly, slower than the molecular consolidation of uniform 2D films, and the hexagonal ordering of the domains measurably improves with waiting time at the deposition pressure. The longevity of the deposited films is extraordinary; the domains become more well-defined with time, presumably as the molecular packing adapts to the two-dimensional solid surface and water evaporates. Nonetheless, the ordering and discrete nature of the aggregates are retained, which reveals a facile route for economical, large-scale nanopatterning of interfaces. This study thus opens up two clear future directions. First in the nanopatterning area, ideally cadmium is avoided unless it is crucial for particular applications, such as for nanodots and properties that are metal-specific. Second, it seems likely that these principles can also be applied to phospholipids and other biologically relevant materials, leading to the possibility of both monodisperse ordered domains and control of the curvature of model membranes.

## Supporting Information Available

Isotherms of 18-MEA and its mixtures with EA on pure water; BAM images of 18-MEA on a neat water subphase; AFM images of 18-MEA deposited from a neat water subphase; FT analysis of AFM images of deposited monolayers of 18-MEA from a Cd^2+^ subphase; long-term domain stability at the air–solid interface; height line profiles from AFM images of drop casted fatty acid films; optical microscopy and supplementary AFM images of drop-casted fatty acid films; BAM images of 18-MEA and mixed fatty acid system 18-MEA:EA; Gibbs free energy comparison and compressional modulus (PDF)
