# Supplementary material for: Unveiling Texture and Topography of Fatty Acid Langmuir Films: Domain Stability and Isotherm Analysis
Source: Langmuir. 2024 May 7;40(20):10468–76. doi: 10.1021/acs.langmuir.3c03501 (PMC11112731; doi:10.1021/acs.langmuir.3c03501)
Supplement: Supplementary file 1 — la3c03501_si_001.pdf [file la3c03501_si_001.pdf]

Electronic Supporting Information

Unveiling Texture and topography of fatty acid Langmuir films: domain stability and isotherm analysis

Erik Bergendal[1], Mark W. Rutland*[1,2,3,4]

Table of contents

- Rights and permissions of adapted isotherms in Figure 2
- Isotherms of 18MEA and its mixtures with EA on pure water
- BAM images of 18-MEA on a neat water subphase
- AFM images of 18-MEA deposited from a neat water subphase
- FT analysis of AFM images of deposited monolayers of 18-MEA from a Cd^{2+} subphase
- Long-term domain stability at the air–solid interface
- Height line profiles from AFM images of drop casted fatty acid films
- Optical microscopy and supplementary AFM images of drop casted fatty acid films
- BAM images of 18-MEA and mixed fatty acid system 18-MEA:EA
- Gibbs free energy comparison and compressional modulus

*Corresponding author

[1] KTH Royal Institute of Technology, School of Engineering Sciences in Chemistry, Biotechnology and Health, Department of Chemistry, Division of Surface and Corrosion Science, Teknikringen 30, 100 44 Stockholm Sweden

[2] RISE Research Institutes of Sweden, Box 5607, SE-114 86 Stockholm, Sweden

[3] School of Chemistry, University of New South Wales, Sydney, NSW 2052, Australia.

[4] Laboratoire de Tribologie et Dynamique des Systèmes, École Centrale de Lyon, 69134 Ecully Cedex, France.

Rights and permissions for adapted isotherms in Figure 1

The data in isotherms in figure 1 have been reproduced as follows.

Gaines 1991:adapted with permission from Ref 53 [1]. Copyright 1991 American Chemical Society.

Kato 1996: adapted with permission from Ref 36 [2]. Copyright 1996 Elsevier B.V

Maaloum 2002: adapted with permission from Ref 37 [3]. Copyright 2002 WILEY-VCH Verlag GmbH & Co. KGaA

Fontaine 2005: adapted with permission from Ref 39 [4]. Copyright 2005 American Chemical Society.

De Viguerie 2011: adapted with permission from Ref 40 [5]. Copyright 2011 American Chemical Society.

Isotherms

With a view to understanding the nature of domain formation mentioned above, reference measurements are performed in systems of the same molecules but where there is no domain formation, due to the rather different headgroup areas, ie on a pure water phase. Monolayers of the well characterised eicosanoic acid (EA, also known as arachidic acid) mixed with its methyl-branched analogue 18-methyleicosanoic acid (18-MEA) were first studied using the Langmuir trough. The isotherms are shown in **Figure S1**, for a pure water subphase where the fatty acid mixture is denoted 18-MEA:EA, with ratios in wt% (leading to a ca 1 % difference in mol%). The isotherm for EA agrees well with what has been reported extensively before,[7,11,47,48] entering a tilted condensed phase at "lift-off"[49], followed by a gradual transition to an untilted condensed phase around 19 Å^2 molecule^{-1}, in agreement with the cross-sectional area of a hydrocarbon chain.[17,50,51] (Note that the isotherms in S1 are

offset laterally to the liftoff value to allow direct comparison of the extent of the compression that would ordinarily be associated with 2D psurface phases. Increasing the 18-MEA fraction leads to a systematically reduced collapse pressure of the monolayer and a diminishment of the extent of the untilted condensed phase, which is only observable for the 25:75 fraction. This suggests a disruptive behaviour to the monolayer by the methyl branch of 18-MEA.

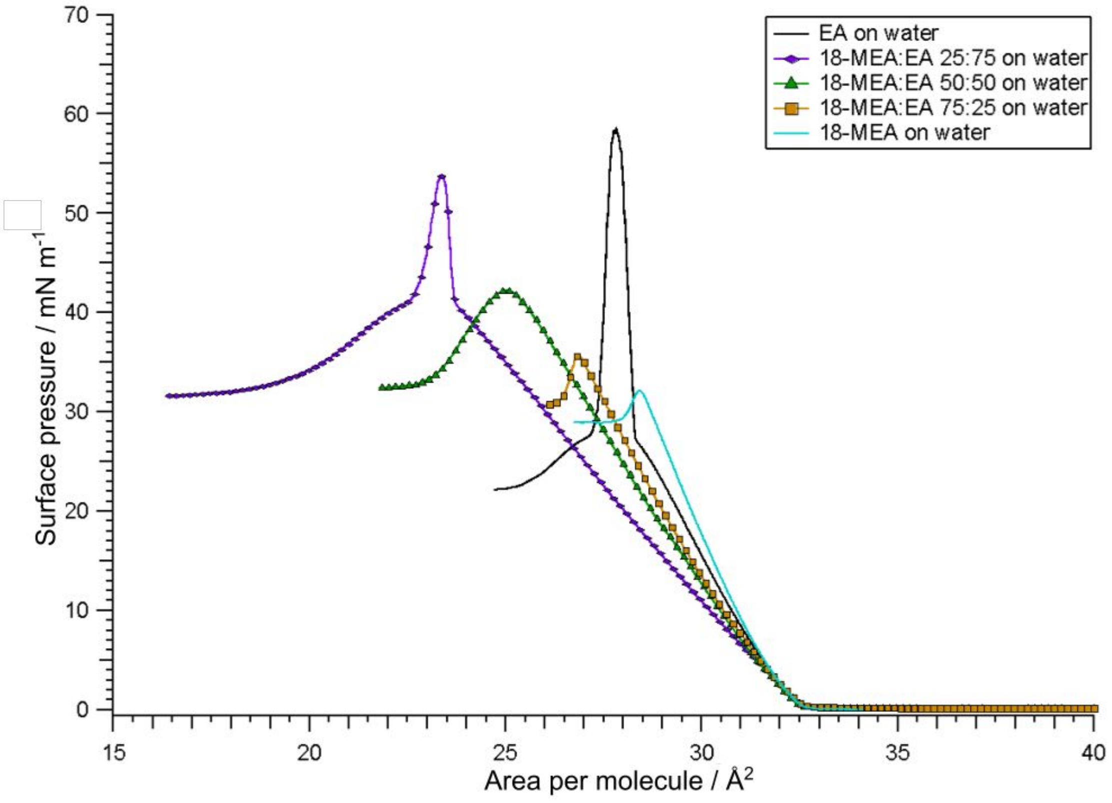

Figure S1. Langmuir isotherms of the saturated long chain fatty acids EA (black line), 18-MEA (blue line), and mixtures of the two with increasing wt% EA given as ratios 18-MEA:EA: 75:25 (squares), 50:50 (triangles), and 25:75 (diamonds). Measurements were performed on a neat water subphase and monolayers were compressed at 9.0 cm^2 min^{-1}. The curves have been

shifted in the x direction to align the "liftoff" and facilitate comparison of the various degrees of compression.

Monolayers of EA deposited using the LB technique from a pure water subphase have been shown to form patchy but homogenous monolayers at the nano-scale,[41] and the coexistence of crystalline domains and disordered monolayer at the micron-scale, observed in-situ with Brewster angle microscopy (BAM).[7,15,52] A BAM image of a floating monolayer of 18-MEA on a neat water subphase is presented in **Figure S2**, showing a foam-like structure of low-density domains at low pressure (0.1 mN m^{-1}), followed by a homogenous monolayer above 5 mN m^{-1}. Films of 18-MEA deposited with the LB technique from a neat water subphase at 0 mN m^{-1}, and subsequently imaged with atomic force microscopy (AFM) are presented in **Figure S3**. The same "foam"-boundaries observed with BAM are observed with AFM, as well as worm-like structuring clearly visible in both height and phase mode AFM imaging, which may reflect the structure in the low density domains. The structuring disappears upon deposition at a higher surface pressure (consistent with BAM images at 5 mN m^{-1}) in the height mode AFM image but is nonetheless conserved, and visible at a higher spatial density in the phase image. This is indicative of crystalline and less ordered regions in the deposited monolayer. The isotherm behaviours of 18-MEA and EA are thus, uncontroversial and mutually indicative of an increased monolayer density with increasing surface pressure.

BAM images of 18-MEA on a neat water subphase

BAM images of 18-MEA on a neat water subphase from foam-like structure at isotherm lift-off at 0.1 mN m^{-1} and a featureless homogenous monolayer above 5.0 mN m^{-1}.

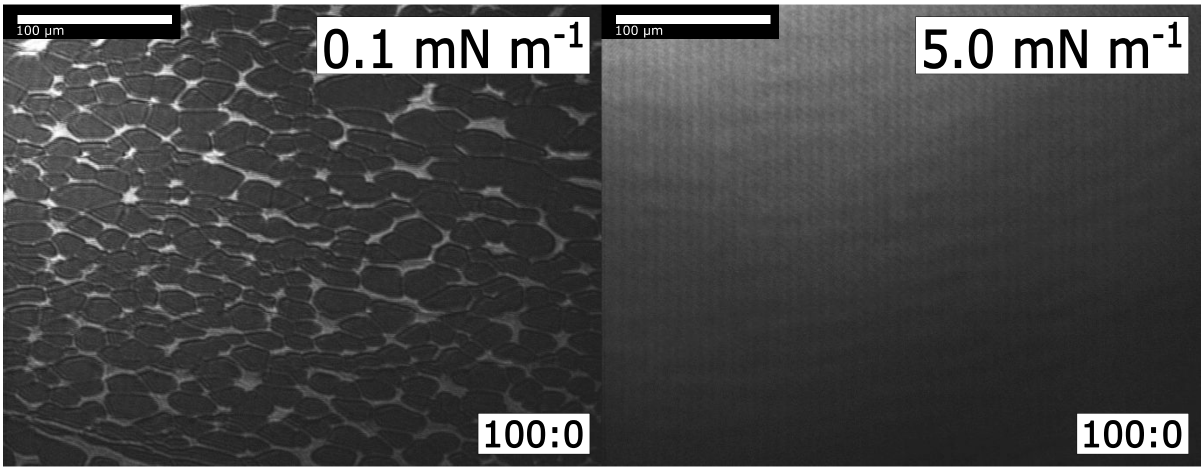

Figure S2 BAM images of 18-MEA on a neat water subphase. Images were taken at monolayer lift-off at 0.1 mN m^{-1} (left) and at 5.0 mN m^{-1} (right). The scale bar to the top left is 100 µm.

AFM images of 18-MEA deposited from a neat water subphase

AFM images of monolayers of 18-MEA deposited onto silicon wafers, deposited at 0 mN m^{-1}, before isotherm lift-off, as well as at 20 mN m^{-1}, are shown in Figure . Results are discussed in detail in the main article.

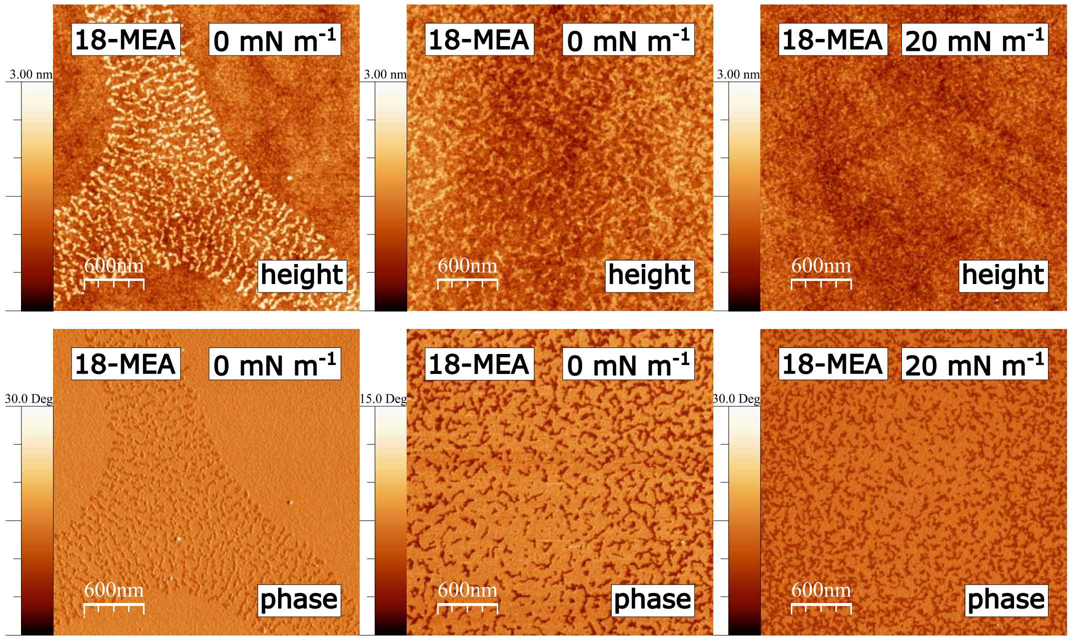

Figure S3 AFM height (top) and phase (bottom) images of 18-MEA deposited on silicon wafers at surface pressures of at 0 mN m^{-1} (left and middle) and 20 mN m^{-1} (right). The height and phase scalebars start at zero nanometre and degrees.

FT analysis of AFM images of deposited monolayers of 18-MEA from a Cd^{2+} subphse

Azimuthal intensity integration was performed using the ImageJ software, [6] for a monolayer of 18-MEA after increasing stabilisation time at the air–water interface before Langmuir–Blodgett deposition. Integration was performed between the same pixel radii for all images. The deposition after 15 minutes show indication of hexagonal ordering with six distinguishable peaks. With increased stabilisation time at the interface, the hexagonal ordering increases, as depicted by the six clear peaks after one and five hours of monolayer stabilisation time at the air–water interface. With increasing time at the interface, there is also an increased multilayering, which observably disturbs the data for the 5-hour deposition. No peak-fitting has been performed to compare the FWHM between the 1-hour and 5-hour depositions.

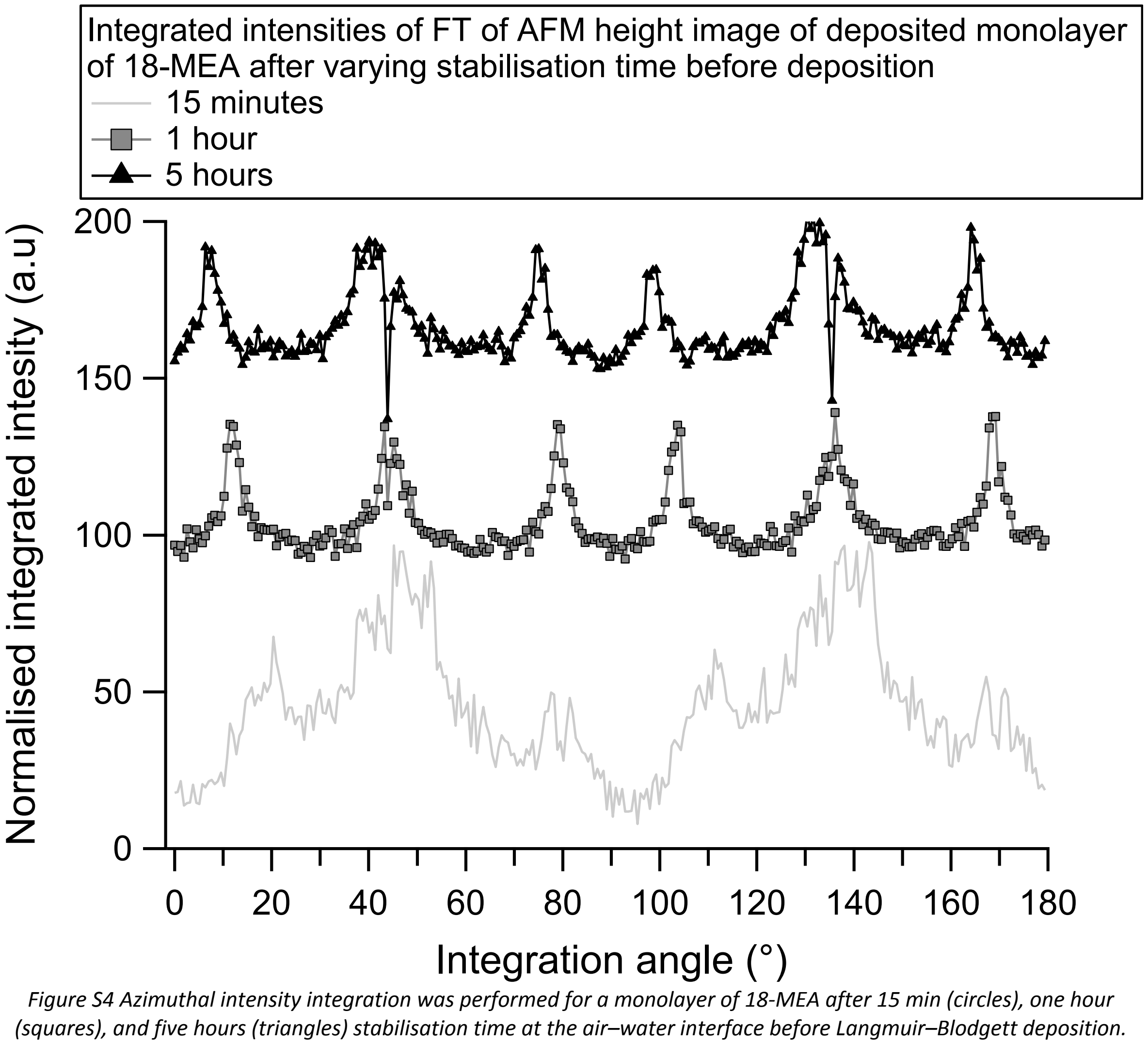

Figure S4 Azimuthal intensity integration was performed for a monolayer of 18-MEA after 15 min (circles), one hour (squares), and five hours (triangles) stabilisation time at the air–water interface before Langmuir–Blodgett deposition.

Long-term domain stability at the air–solid interface

Figure shows AFM height images of deposited monolayers of 19-MEA, imaged almost 2 years after deposition, and storage in an air-tight container in ambient conditions. The domains formed at the air–water interface are stable to transfer to a solid support, and are here shown to demonstrate long-term stability at the air–solid interface. The FT insets in Figure demonstrate a characteristic size of the domains at the air–solid interface.

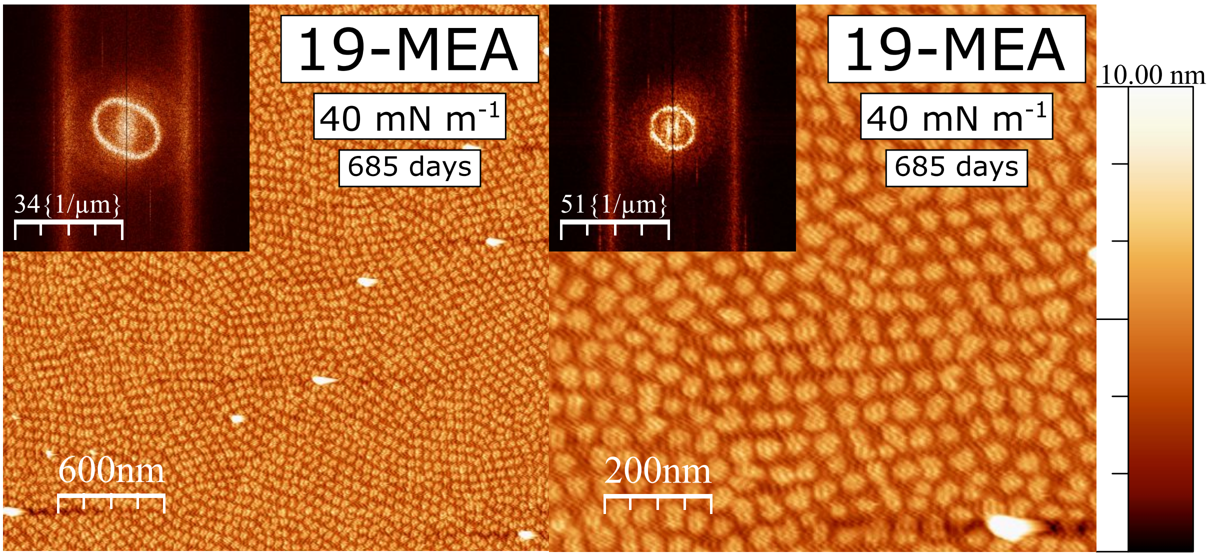

Figure S5 AFM height imaging showing the long-term stability of monolayers of 19-MEA deposited on silicon substrate from a 0.1 mM Cd^{2+}-subphase at pH 6.0. Imaging was performed nearly 2 years (684 days) after deposition. FT insets indicate a characteristic domain size at the air–solid interface.

Height line profiles from AFM images of drop casted fatty acid films

AFM height images of drop-casts made on dry silicon wafer are shown in Figure for 18-MEA:EA 100:0 (left), 50:50 (middle), and 0:100 (right). Black and blue line-profiles are shown under each respective image.

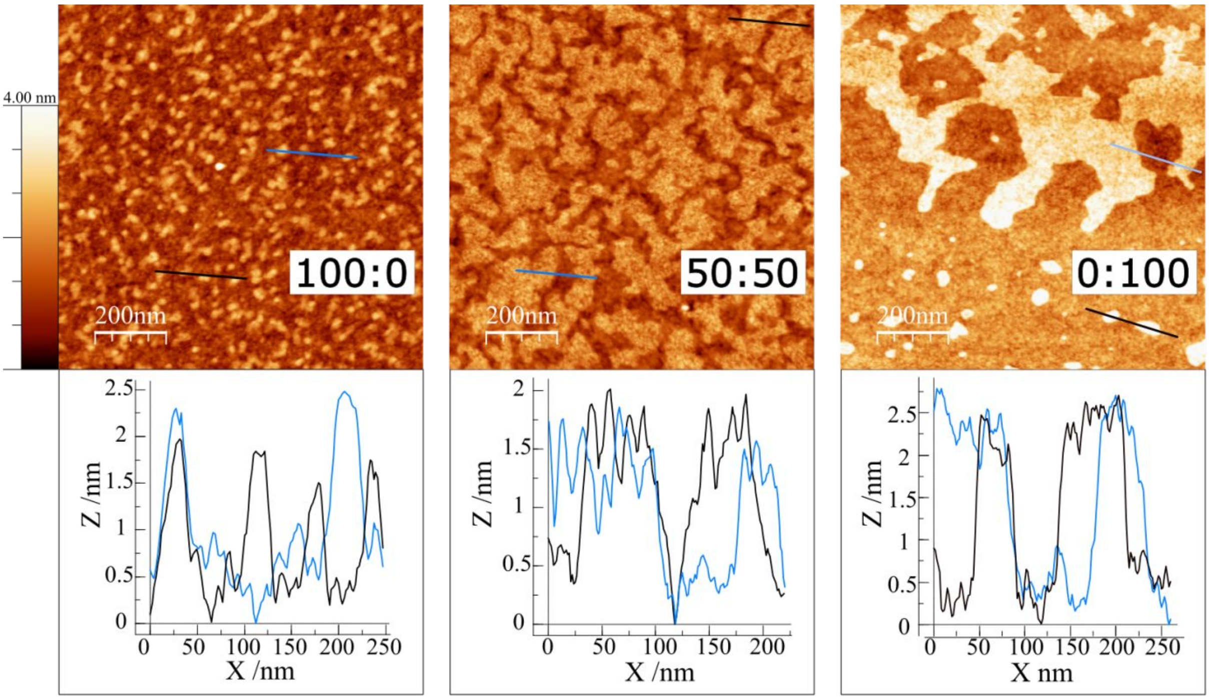

Figure S6 AFM height images of drop-casted 18-MEA:EA 100:0 (left), 50:50 (middle), and 0:100 (right). The amplitude scale-bar shown to the right is representative for all images, and starts at zero nm. Black and blue line-profiles are presented under each respective image.

Optical microscopy and supplementary AFM images of drop casted fatty acid films

Optical microscopy of micrometre-sized crystallites resulting from drop-casting a 1.0 mg mL^{-1} fatty acid chloroform solution of 18-MEA:EA 50:50 on a dry silicon wafer.

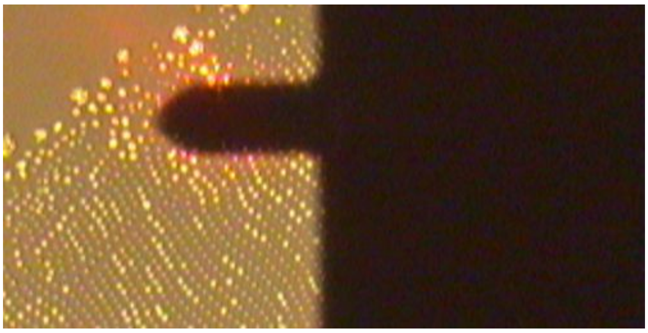

Figure S7 Optical microscopy image of drop-casted 18-MEA:EA 50:50. AFM-cantilever is approximately 110 µm long.

Crystallites observed by optical microscopy in Figure S7 were further imaged by AFM in Figure . The crystallites observed in 50:50 (bottom image) show two distinct morphologies: one type which is approximately 50 nm in height over several micrometres, and one more disordered type of around 250 nm height. This could possibly be a result of phase separation between the branched and the straight chain fatty acids during chloroform evaporation. This could be investigated with nanoscale Fourier transform infrared spectroscopy (Nano-FTIR). [7] Higher resolution AFM-images of the drop-

casts are shown in Figure S9, simultaneously showing the micron-scale crystallites and the varying monolayer morphology between them.

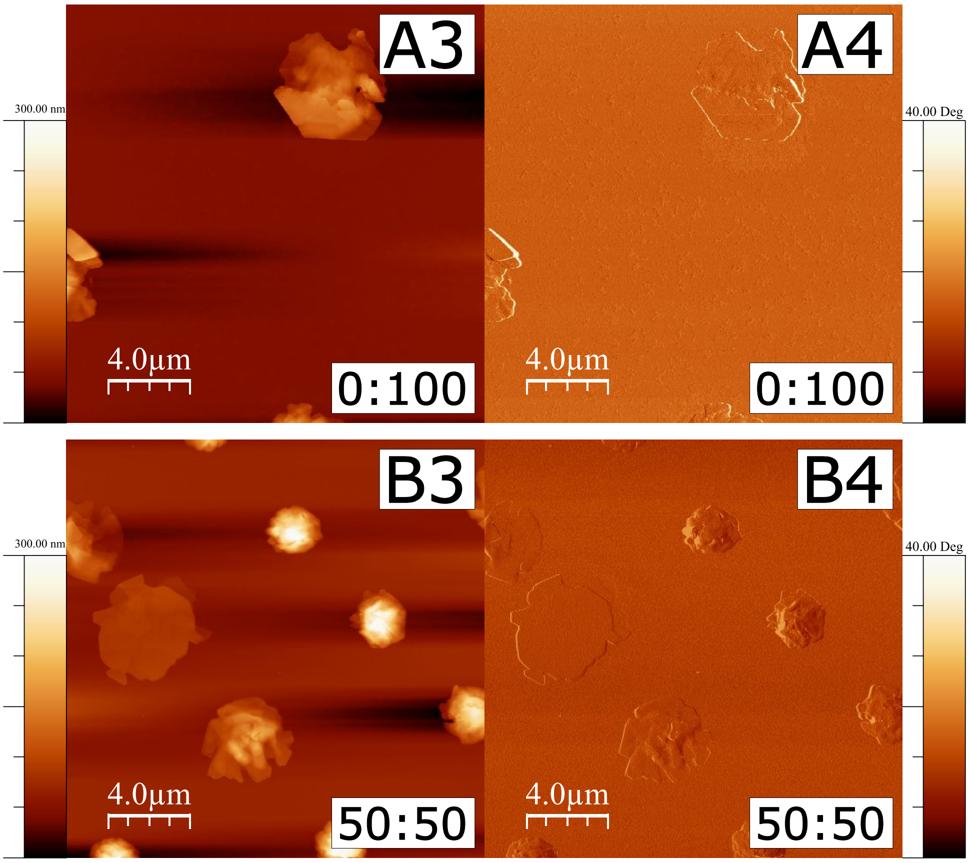

Figure S8 AFM height (left) and phase (right) images of drop-casted 18-MEA:EA 0:100 (top) and 50:50 (bottom). Height and phase modulation scale-bars start at zero nanometre and degrees, respectively.

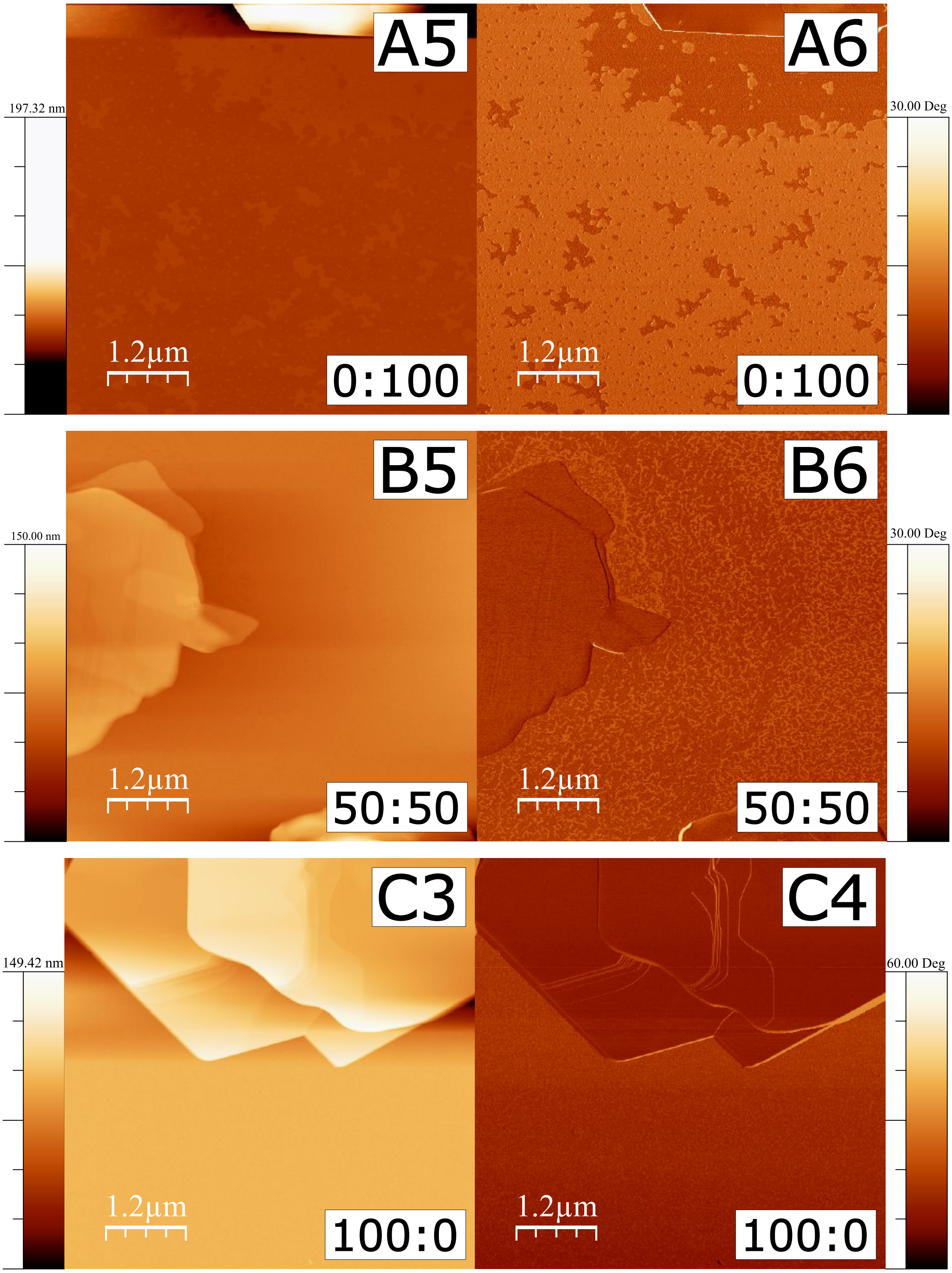

Figure S9AFM height (left) and phase (right) images of drop-casted 18-MEA:EA 0:100 (top) and 50:50 (middle), and 100:0 (bottom). Height and phase modulation scale-bars start at zero nanometre and degrees, respectively.

BAM images of 18-MEA and mixed fatty acid system 18-MEA:EA on a Cd^{2+} subphase

BAM images of 18-MEA:EA are shown in Figure . Measurements were performed on a 0.1 mM Cd^{2+} subphase at pH 6.0, and images were captured at isotherm lift-off at a surface pressure of 0.3 mN m^{-1}. The bright areas represent monolayer whereas the black regions shows the lack of reflection of *p*-polarised light at the Brewster angle of 53.1°.

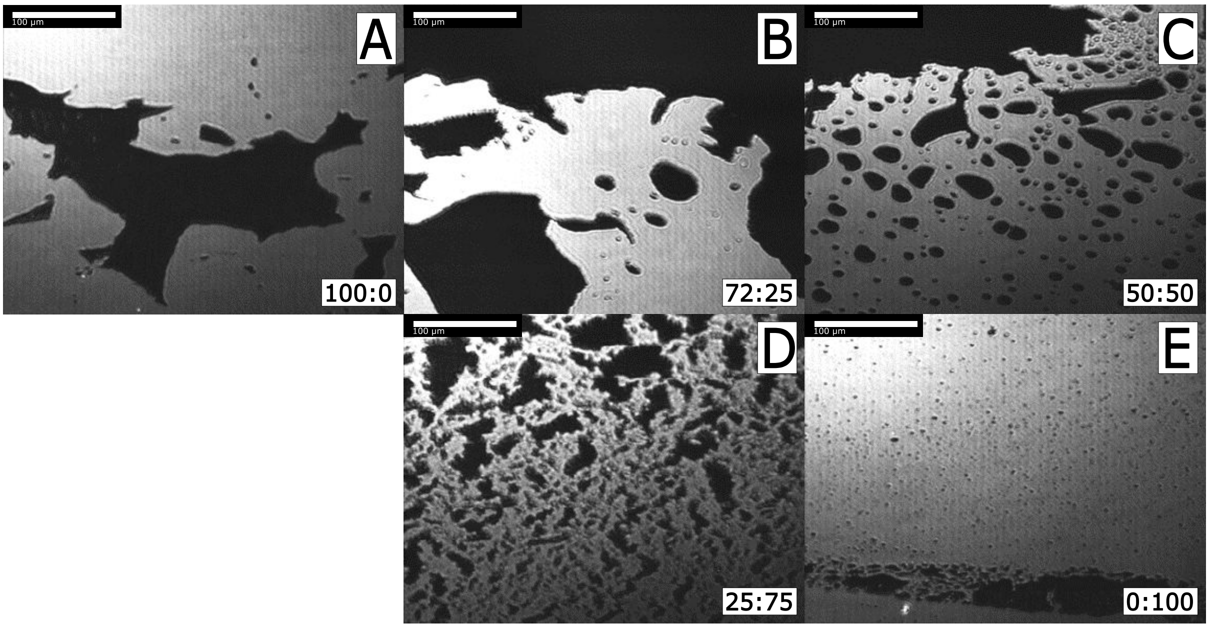

Figure S10 BAM images of 18-MEA:EA taken at isotherm lift-off at 0.3 mN m^{-1} on a subphase of 0.1 mM Cd^{2+} at pH 6.0. The bright areas represent monolayer reflection and the black areas is the lack of reflection of p-polarised light from water subphase at the Brewster angle. The scalebar represents 100 µm.

Above isotherm lift-off, all monolayers showed identically featureless homogenous monolayers as are presented for 18-MEA in Figure S1. Any domain formation in the monolayer is on a length-scale not resolvable by BAM.

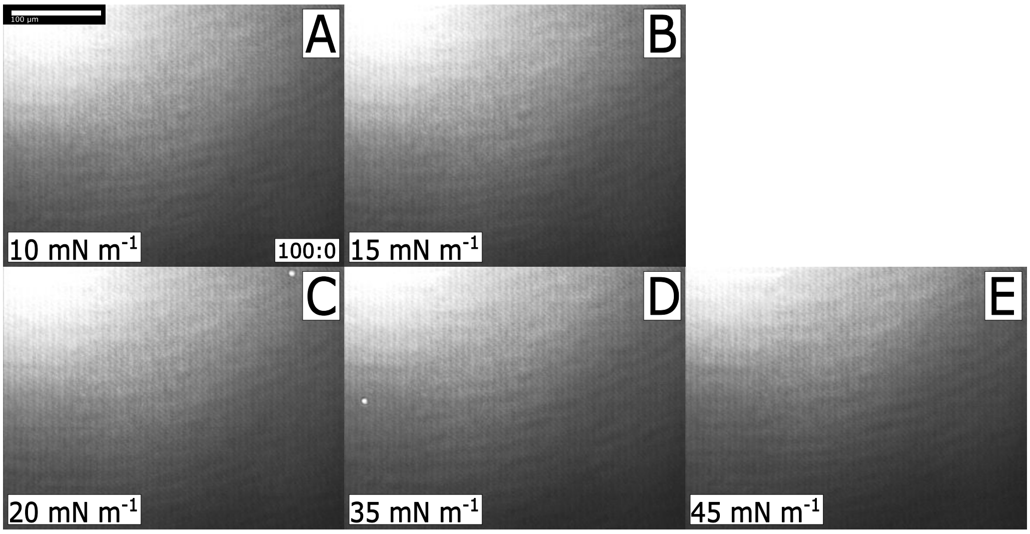

Figure S1 BAM images of a monolayer of 18-MEA taken at increasing surface pressure on a subphase of 0.1 mM Cd^{2+} at pH 6.0. Measurements were performed at the Brewster angle for water, the reflection is thus from a featureless homogenous floating monolayer. The scalebar represents 100 µm.

AFM images of deposited monolayers of mixed fatty acid systems 18-MEA:EA, adapted from

Domain formation in fatty acid monolayers of 18-MEA:EA, deposited on silicon wafers from 0.1 mM Cd^{2+} subphase at pH 6.0. The images show a variety of domain shapes and sizes determined from the ratio of branched and straight chain fatty acid. The images are reproduced from ref 43.

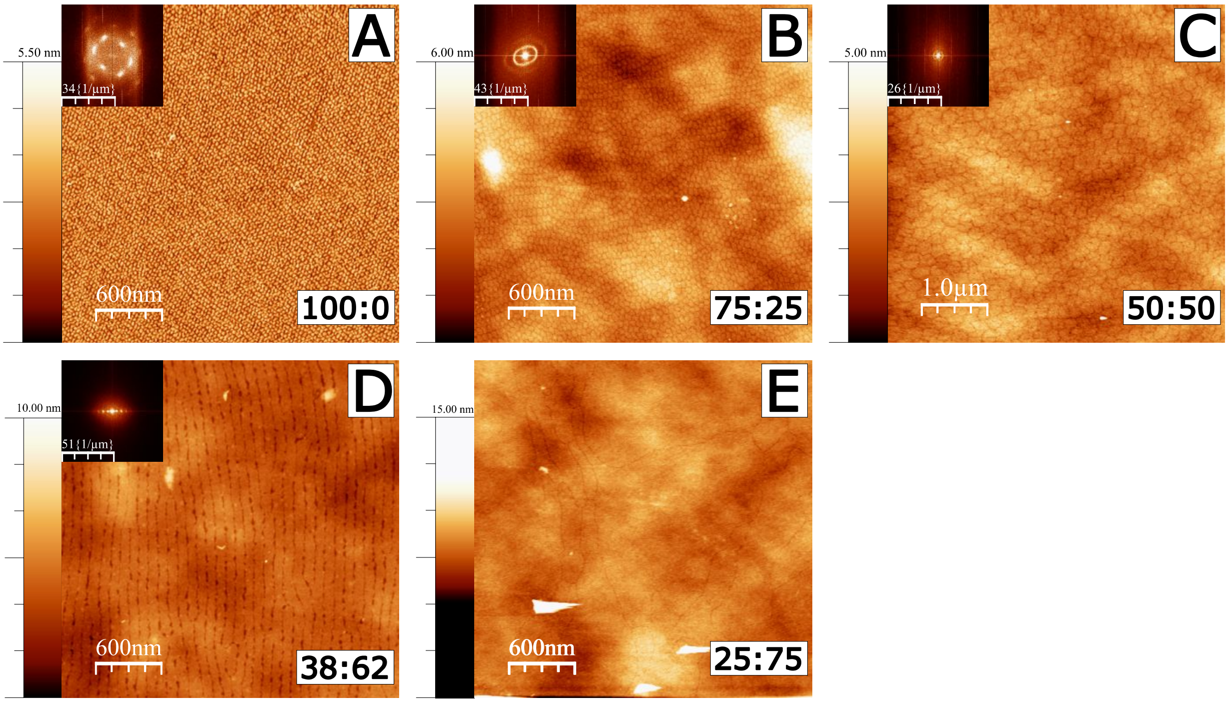

Figure S12AFM height mode imaging of 18-MEA:EA monolayers formed at the air–water interface and deposited on silicon wafers. Surfactant ratios stated in wt. % for 18-MEA:EA are shown in the bottom right for each deposition. A Fourier transform is shown as inset to the top left of each image, except for the 25:75 mixture where no correlated surface structure is observed. Height variation scalebars all start at zero nanometre. All depositions were made at 20 mN m^{-1}.

Gibbs free energy comparison and compressional modulus

The domains formed by branched long chain fatty acids have been concluded to three-dimensionally texture the air–water interface. This induces a height-modulation of the water surface, observed with neutron reflectometry. A simple relationship between the previously reported domain-size measured by AFM on deposited monolayers and the appurtenant height-modulation induced from neutron reflectometry, is used to calculate a real interfacial area at the air–water interface, compared to the apparent area deduced from pressure–area isotherms. Form this, the energy-penalty associated with the presented area-difference is calculated at a certain surface pressure. The calculated energy penalty from domain-forming mixed monolayers is then compared to the linear combination deduced from its components 18-MEA, and EA.

The compressional modulus for monolayers of EA, 18-MEA, and mixtures of the two was calculated from pressure–area isotherms, and is presented in Figure S2. The data-sets are shown as lines between points and markers at every 5 data point, except for EA where a dashed line connects the data points. Due to the mechanical constraints of the Langmuir trough hard-ware, the data was not collected as a smooth line, but as a step-function. Thus, a 20-point moving average was used to smooth the data in the software Igor Pro (WaveMetrics, United States), with the build-in box-smooth operation.

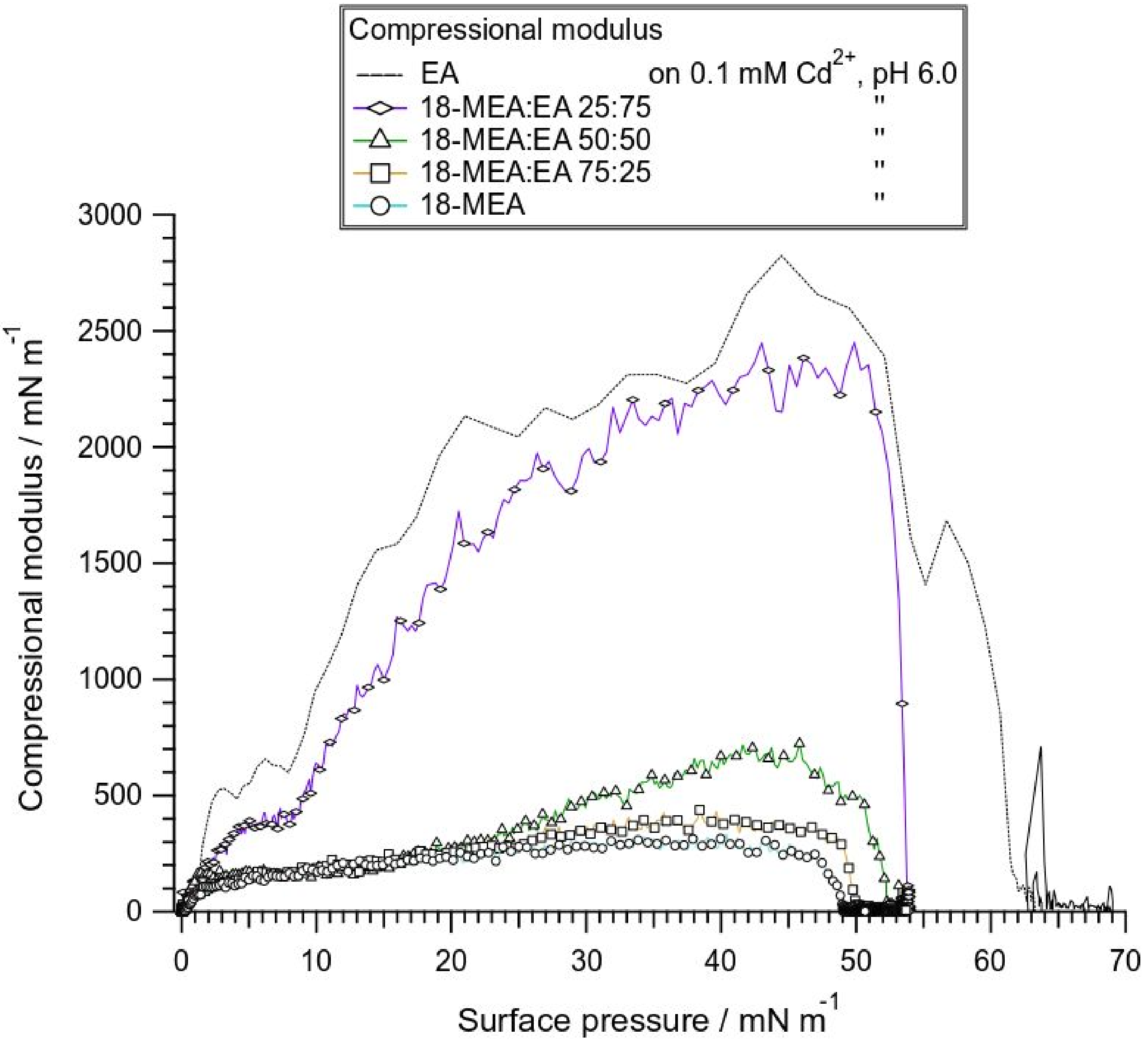

Figure S2 Compressional modulus calculated from isotherms of 18-MEA:EA (100:0 – circles, 75:25 – squared, 50:50 – triangles, 25:75 – rhobi, 0:100 – dashed line) collected on a 0.1 mM Cd^{2+} subphase at pH 6.0.

References

1. Gaines, G.L. Surface Activity of Semifluorinated Alkanes: F(CF2)m(CH2)nH, *Langmuir* **1991** *7* 3054–3056

2. Kato, T.; Kameyama, M.; Kawano, M. Two-dimensional micronodule structure in monolayers of a partially fluorinated long-chain acid observed by atomic force microscopy, *Thin Solid Films*. **1996** *273*, 232–235..

3. Maaloum, M. ; Muller, P. ; Krafft, M.P. Monodisperse Surface Micelles of Nonpolar Amphiphiles in Langmuir Monolayers, *Angew. Chemie - Int. Ed.* **2002,** *41,* 4331–4334.

4. Fontaine, P. ; Goldmann, M. ; Muller, P. ; Fauré, M.C. ; Konovalov, O. ; Krafft, M.P. Direct evidence for highly organized networks of circular surface micelles of surfactant at the air-water interface, *J. Am. Chem. Soc*. **2005** *127*, 512–513..

5. De Viguerie, L.; Keller, R.; Jonas, U.; Berger, R.; Clark, C.G.; Klein, C.O.; Geue, T.; Müllen, K.; Butt, H.J.; lassopoulos, D. V. Effect of the molecular structure on the hierarchical self-

assembly of semifluorinated alkanes at the air/water interface, *Langmuir*. **2011,** *27*, 8776–8786.

6. Schneider, C. A. ;Rasband W. S.;. Eliceiri, K. W *Nat. Methods*, **2012**, *9*, 671–675.

7. Huth, F. ; Govyadinov, A.; Amarie, S.; Nuansing, W. ; Keilmann F.; Hillenbrand, R. *Nano Lett.*, **2012**, *12*, 3973–3978.
